# Drawing conclusions: Confessions of a scientist who thinks better with a pencil in hand

**DOI:** 10.1038/s44319-026-00792-7

**Published:** 2026-05-02

**Authors:** André Schneider

**Affiliations:** Department of Chemistry, Biochemistry and Pharmaceutical Chemistry, University of Bern, Freiestrasse 3, CH-3012 Bern, Switzerland

**Keywords:** History & Philosophy of Science, Methods & Resources

## Abstract

Drawing to understand or explore concepts, complex relationships or cells enhances both creativity and analytical thinking in science. The same process also helps to design clearer, more effective conceptual figures for publications.

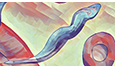

If you study life sciences, your training will expose you to mathematics, chemistry, physics, computer science and much more. You will learn how to formulate hypotheses and design experiments and controls to test them. And by the end of your bachelor’s or master’s degree, you will have a good knowledge of the many tools in your field.

What is often overlooked, however, is that science is not just a technical profession but a profoundly creative endeavour. Science and art, though they may seem worlds apart, share deep roots. Leonardo da Vinci sketched anatomy with as much artistry as precision, and Robert Hooke turned what he saw through his microscope into beautiful drawings. Even today, this connection remains strong and can be quantified: Nobel laureates are nearly three times more likely than other scientists to pursue artistic hobbies (Root-Bernstein et al, 2008).

Science and art, though they may seem worlds apart, share deep roots.

Yet, few scientists are ever explicitly taught how to be creative. Luckily this is beginning to change. A particularly exciting example is the Night Science podcast by Itai Yanai and Martin Lercher, which does not explore scientific results, but the creative processes behind them. Listening to their conversations—along with the fact that I recently retired from my position as a biochemistry professor—inspired me to reflect on my own creative process.

Like many scientists, an idle mind during a hot shower, an “incubation” in a sauna, or jogging and cycling, greatly helped me to have new ideas and to connect in unexpected ways what previously seemed to be unrelated facts. But for me, one catalyst stood out: drawing. My creative process often began with unconscious doodling, which sometimes evolved into more intentional sketches inspired by my own research. Over time, I realized that these drawings not only sparked ideas but also sharpened how I look at figures in scientific publications.

## How it all started

Whenever I had a pen and paper in front of me, I would doodle. It wasn’t a choice, but a compulsive urge. This habit began in high school. My history teacher once tried to ban me from doodling during class. It worked for a short time only before my hand resumed its restless scribbling. Annoyed by my behaviour, he tried to catch me off guard and asked me a difficult question. To his—and my—surprise, I knew the answer. From then on, he gave me official permission to doodle during his lectures. That was when I first realized that doodling was not merely a distraction, but helped me to focus.

Much later, I discovered research showing that doodling can indeed improve concentration (Andrade, 2010). The other seemingly paradoxical effect of doodling is that it can induce this state of a wandering or idle mind that enables creativity. I also always scribbled during formal Faculty meetings as my colleagues can testify. While this may have helped me to concentrate, the subject of my focus only rarely had a connection to agenda points of the meeting. Many of my doodles started with a schematic drawing of my favourite cell or molecules, others were entirely abstract. Most often though it was a mixture of both. Over time, I noticed a pattern: alternating between deep focus and semi-automatic doodling put me in my most creative state. That is why I began saving my scribbles instead of discarding them. Revisiting them later, I often found the seeds of ideas for larger drawings that captured concepts from my lab’s research more vividly than words alone could.

The other seemingly paradoxical effect of doodling is that it can induce this state of a wandering or idle mind that enables creativity.

I would like to share three of these drawings now and reflect on how creating them helped sharpen both my scientific thinking and creativity. It is no accident that all of them feature the parasitic protzoan *Trypanosoma brucei*. After more than thirty years of working with these cells, it’s hard not to develop an almost intimate relationship with them. If you’re a scientist, I think you will know what I mean.

## Form follows function

**Figure 1:**
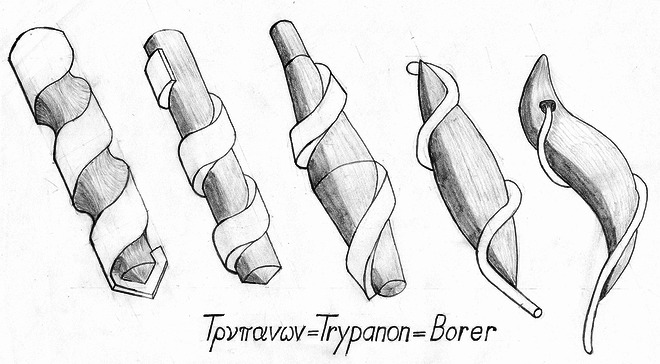
Form follows function. The pencil drawing shows different stages of an imagined metamorphosis of a drill into a cell of the unicellular parasite *T. brucei*. It is an artistic interpretation of the etymology of the genus name “Trypanosoma” which derives from the old Greek word “trypanon” meaning “borer” in English, alluding to the borer-like shape of trypanosomes.

The first image was inspired by the word “*Trypanosoma”* itself (Fig. 1). It derives from the Greek *trypanon* (drill) and *soma* (body) and was introduced by David Gruby in 1843 to describe the organism’s corkscrew-like motion (Gruby, 1843). I wanted to visualize this etymology by drawing an imagined metamorphosis from a drill into a trypanosomal cell. However, when I tried to draw the final form, a simple trypanosomal cell, I ran into problems. Unlike verbal descriptions, drawing allows no vagueness, every line demands a decision. I realized, uncomfortably, that I did not truly know what a trypanosome looks like.

That failure was instructive. Trypanosomes have no single, fixed shape. They are flexible and highly motile, and it is their movement, rather than a defined geometry that inspired their name. The most obvious features of a non-dividing trypanosome are the cell body and the single flagellum that is laterally attached to it. However, the precise shape of the cell body is surprisingly elusive.

To resolve this, I turned to the literature and studied numerous pictures obtained by light and electron microscopy (EM). Scanning EM images proved most useful. After many attempts, I settled on a sequence of shapes to morph the drill into the cell body of the trypanosome: a cylinder, two joined truncated cones, and a spindle. The latter then transformed into an elongated curved spindle which was the best approximation of the trypanosomal cell body I could draw. While these transitions were mainly guided by aesthetics, the drawing began to suggest how biology might work. The transformation of the drill’s surface into a flagellum implied that its attachment to the cell body and tension, and not simply static geometry, sculpt the cell. This intuition reflects reality. Trypanosomal shape arises from the interplay between the attached flagellum and the cell body (Sunter and Gull, 2016). It is constrained by a microtubular corset beneath the membrane and inherited through cell division. Gruby could not know this, his microscope was not good enough, but trypanosomes not only move like a drill, they are shaped like one. By drawing, I moved beyond illustration into speculation. I was wondering, how did this shape evolve? Might ancestral trypanosomes once have resembled the simpler, transitional shapes along my imagined path?

By drawing, I moved beyond illustration into speculation.

## (De)constructing trypanosomes

**Figure 2:**
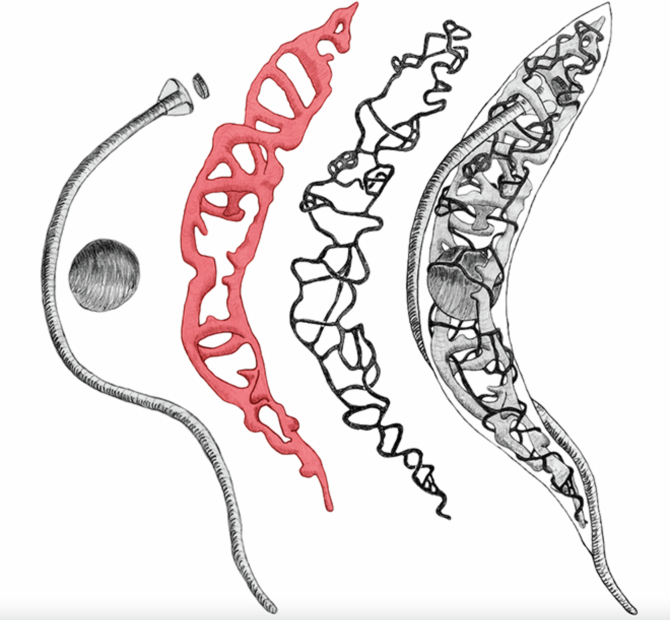
(De)constructing trypanosomes. This pencil dawing depicts, from left to right: (i) Nucleus, mitochondrial genome and the flagellum, (ii) the single mitochondrion (red), (iv) the endoplasmic reticulum (ER) of a *T. brucei* cell. The picture on the very right is composite image combining all three structures in a complete *T. brucei* cell.

Exploded-view technical blueprints have always fascinated me. They reveal, in a single image, how separate parts form a coherent whole and provide insights into how the depicted object might function. In Fig. 2, I emulated this style for the trypanosomal cell to highlight its distinctive features.

Guided by both aesthetics and my long-standing interest in the mitochondrion, I deconstructed the cell into three building blocks: DNA-containing structures and the flagellum; the single mitochondrion; and the endoplasmic reticulum (ER). Finally, I created a composite image combining all three parts. Sketching the first two parts immediately sharpened my thinking. The act of first separating and then reassembling them highlighted a defining feature of trypanosomes: the single mitochondrion containing the highly condensed single-unit mitochondrial genome that is physically linked to the flagellar basal body.

The ER, however, resisted easy depiction. As there were no 3D reconstructions available at the time, I therefore examined immunofluorescence images and EM cross sections. From these, I inferred that both the mitochondrion and the ER form extensive cytosolic networks, that are often in close contact, with the ER displaying finer branches. Because the data were incomplete, I could not simply illustrate what these images showed but had to develop my own spatial interpretation of the ER architecture. In doing so, drawing became a conceptual tool, it helped me to understand how the ER might weave through the cell and where it might touch the mitochondrion.

This visual reconstruction also prompted me to deeply think about how form and function might be connected. In yeast and mammals, ER–mitochondrion contact sites mediate lipid transfer and mark mitochondrial fission sites to ensure proper distribution of mitchondrial genomes during cytokinesis (Murley et al, 2013). But as I completed the full-cell drawing, the widespread distribution of contact sites in trypanosomes became apparent. That spatial insight suggested they were unlikely to control inheritance of the single mitochondrial genome, which is confined to a small region of the organelle (Aeschlimann et al, 2023). Thus, the exploded-view drawing did more than depict trypanosome-specific features. It stimulated new conceptual insights into how mitochondrial genome segregation in trypanosomes might differ from that in other eukaryotes.

## A fantastic voyage

**Figure 3:**
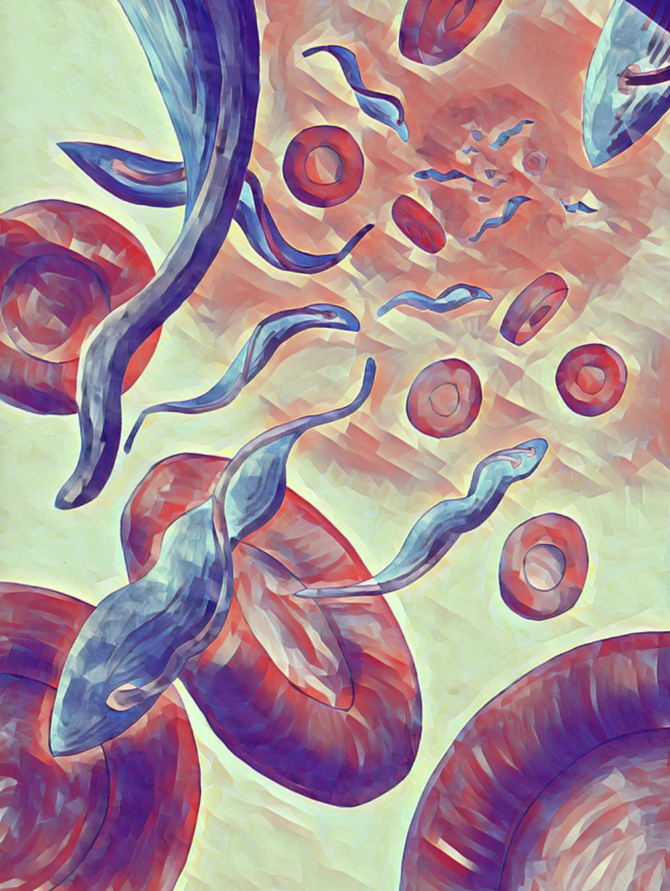
A fantastic voyage. The picture depicts an artistic rendering of *T. brucei* cells with erythrocytes in a human bloodvessel. *T. brucei* is transmitted by the Tsetse fly and causes sleeping sickness in humans. The iginal pencil and watercolour painting was processed with the free online foto editing software Lunapic using the polygons tool.

Have you seen the 1966 science fiction movie “Fantastic Voyage”, where a team of scientists in a submarine are shrunk to microscopic size and injected into the bloodstream of a human patient with the mission to remove a blood clot in the brain? In molecular and cell biology, most methods are indirect because the objects of study are too small, too dynamic or inaccessible to be observed directly. Wouldn’t it be cool to be one of the scientists in this movie and to be able to directly observe cells and biochemical processes in real time from such a perspective? Just imagine how much we could learn from that. Unfortunately, this works only in a Hollywood movie. However, drawing allows the artist to impose a specific viewpoint on the observer. This was what I tried to do in the third picture (Fig. 3). I imagined I would be about 1 μm long and swim with trypanosomes in a small blood vessel of a patient. To make the picture more immersive, I chose an extreme perspective: nearby trypanosomes and red blood cells partially obstruct the field of vision of the viewer, enhancing the sense of presence within the scene.

Although I did not set out to illustrate a specific scientific concept, the process of sketching and painting prompted me to think about scale in biology. With about 25 μm in length, a trypanosome would appear to me close to 50 m long, a bit bigger than a blue whale. Yet, I was surprised to learn that, while I would be able to see individual ribosomes, their diameter would span 6 cm only and a haemoglobin molecule in red blood cells would only be 1 cm wide.

Although I did not set out to illustrate a specific scientific concept, the process of sketching and painting prompted me to think about scale in biology.

Revisiting the picture five years later, while writing this essay, shifted my perspective in yet a different way. It is one of the very few drawings in which I depicted trypanosomes inside their human host, where they cause sleeping sickness. I have always seen myself as a basic scientist pursuing fundamental biological questions, not primarily as a parasitologist combating disease. Yet, the image reminded me that the trypanosomes we studied in my lab had been cultured in vitro for many years in rich media, far removed from their natural environment. Thus, what we were doing in the lab was akin to studying elephants in a zoo, where much of their natural behaviour is known to be altered. The drawing thus also became a humbling reminder of the implicit limitations of our research.

## The value of making figures from scratch

**Figure 4:**
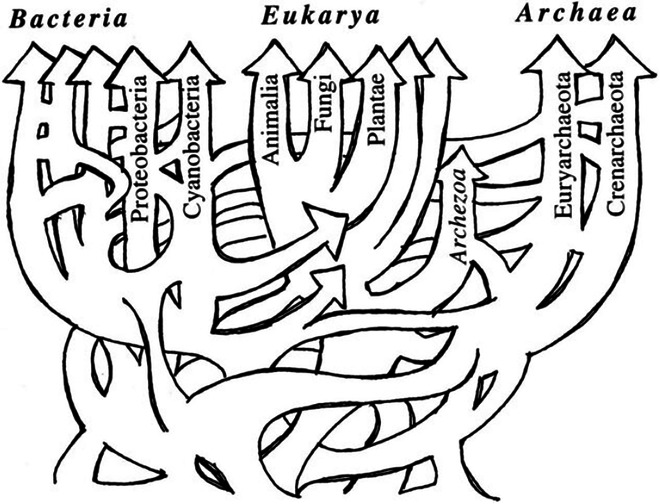
The entangled tree hand-drawn by Ford Doolittle. Phylogenetic tree illustrating the important role horizontal gene transfer plays in evolution. Reproduced from Doolittle (1999) with permission by Science/AAAS.

Figures in life sciences publications come in two flavours: the ones showing data and the rarer ones, often found at the end of a paper or in review articles, that present a model of how a mechanism or a pathway may function. When done well, such conceptual figures can add substantial value to a paper. A striking example is Ford Doolittle’s hand-drawn, reticulate phylogenetic tree, which to his surprise was published unedited alongside his review article (Fig. 4; Doolittle, 1999). A single glance at this picture suffices to convey the profound implications extensive horizontal gene transfer has for a “natural” classification of organisms.

**Figure 5:**
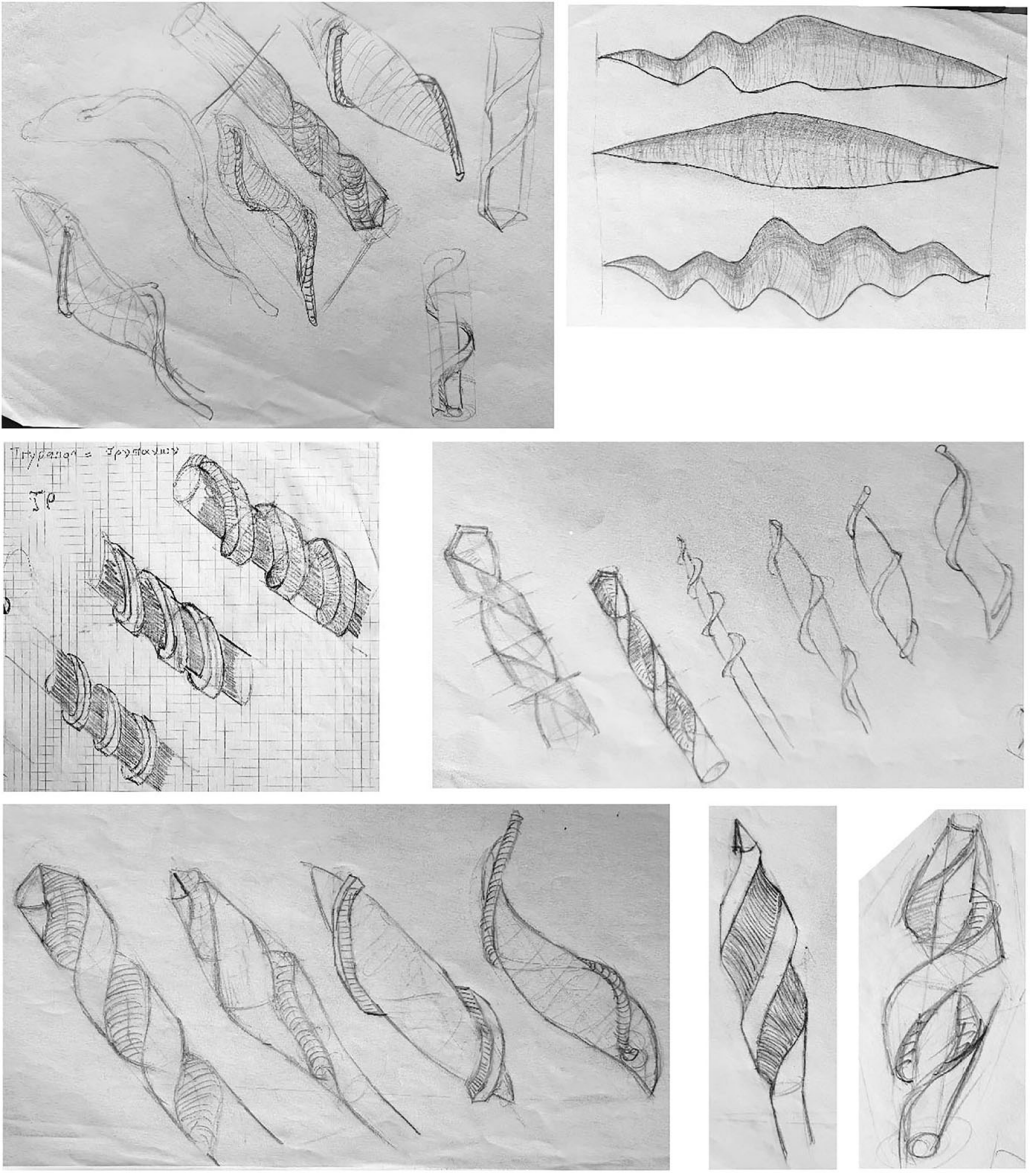
Putting an idea on paper requires many preliminary sketches. Small selection of preliminary sketches that were used to work out the details and optimize the metamorphosis drawing shown in Fig. 1.

My drawings have changed how I approach such conceptual figures. Similar to how professional artists work, producing these science-inspired pictures requires many preliminary sketches. Some of those for the metamorphosis drawing (Fig. 1) are shown in Fig. 5 to highlight the iterative process of refining an idea. In these early stages, ambiguity is an asset rather than a flaw, it enables unexpected connections and alternative representations to emerge. It turns out that the same process is also immensely helpful when drawing conceptual figures.

In these early stages, ambiguity is an asset rather than a flaw, it enables unexpected connections and alternative representations to emerge.

But what makes a good conceptual figure? Above all, it must communicate a clear idea. This works best when every single element, such as geometric shapes, colours, arrows and other symbols, serves a defined role in expressing that idea. Anything that does not contribute to the message becomes a distraction and should be removed. Sounds simple, right? In practice, this is much harder than it sounds. When I revisited published figures I designed myself, I realized that I often had failed in this task.

For this reason, I recommend starting conceptual figures early in a project, long before a paper is written. Designing figures from scratch takes time, but that time is well spent. The main benefit is not just the final perfectly designed figure but the iterative design process itself. Translating experimental results into a visual model forces you to make choices. Which features of a molecule, pathway or cell are essential to convey the message? Which features should be omitted because they are distracting? These decisions require both analytical thinking and imagination.

Sharing early versions is equally important. Show your figures in seminars, progress reports and to anyone willing to comment. It is my personal experience, that the act of designing figures triggers an almost obsessive reflection on what the data actually mean. It often exposes gaps in a model and sometimes provides ideas for new elegant experiments. Importantly, specialized drawing programs now allow to digitally design conceptual figures: creating them does not require much artistic talent but stringent logical thinking and visual consistency.

Many institutions now subscribe to the scientific illustration software BioRender, which provides thousands of ready-made icons and templates. Its popularity is easy to understand as it allows users to produce professional-looking figures very quickly. Yet, the results often look superficial—all figures designed with it look strikingly similar. The abundance of icons it provides can tempt users to hide simple concepts behind a forest of symbols and icons.

The deeper drawback, copyright issues aside, is that such tools often bypass the creative and intellectual process that figure design can stimulate. When everyone relies on the same library of icons, often of inconsistent styles, figures become bland and much opportunity for genuine visual thinking is lost. Designing a clear conceptual figure is not merely a technical step in preparing a paper. It is part of the scientific process itself. And like good science, it takes time. There is no free lunch.

Designing a clear conceptual figure is not merely a technical step in preparing a paper. It is part of the scientific process itself.

## Concluding remarks

I am a highly visual thinker. When I reflect on scientific concepts, I see images in my mind, often intricate and rich in detail. Yet these images are fleeting. I can only grasp fragments of them for a fraction of a second, and whenever I try to focus on a specific detail, it dissolves.

Looking back, I realize that drawing gradually evolved from a simple pastime into an integral part of my scientific process. What I was trying to do, in essence, was to capture on paper something I already knew, something that existed in a somewhat liquid form as a kind of visual intuition. Intuition is a difficult concept to define, and its meaning varies across disciplines. Still, there is broad agreement that intuition and creativity are closely intertwined. In my case, doodling and drawing became a way to access these visual intuitions and, through them, to trigger my creativity. There is also something important about the tactile experience itself. Using a pencil, feeling the slight resistance of the paper when drawing, an almost anachronistic practice in a digital age, helped to catalyse the process.

The original meaning of the verb “to draw” is “to pull out”. And indeed, for me drawing a picture is to pull out a mental image onto paper. But there is another aspect. As a scientist I also need to draw conclusions: to pull out and formulate what can be learned from experimental results. Thus, “drawing pictures to draw conclusions” is a fitting summary of the creative process I describe in this essay.

… “drawing pictures to draw conclusions” is a fitting summary of the creative process I describe in this essay.

Finally, some of my drawings, including the three shown in this essay, eventually appeared on the covers of scientific journals. Seeing them there was not only a personal pleasure but also offered a nice way to communicate our scientific work to a wider audience.

## Supplementary information

Peer Review File
