# Supplementary material for: Drawing conclusions: Confessions of a scientist who thinks better with a pencil in hand
Source: EMBO Rep. 2026 May 2;27(11):2845–9. doi: 10.1038/s44319-026-00792-7 (PMC13260322; doi:10.1038/s44319-026-00792-7)
Supplement: Supplementary file 1 — Peer Review File [file 44319_2026_792_MOESM1_ESM.pdf]

EMBO reports

Drawing conclusions - Confessions of a scientist who thinks better with a pencil in hand

André Schneider

Corresponding author(s): André Schneider (andre.schneider@unibe.ch)

Review Timeline:

Submission Date:	8th Apr 26
Accepted:	24th Apr 26

Editor: Holger Breithaupt

Transaction Report: This article was editorially reviewed.
